# Age assessment of unaccompanied foreign minors: an analyses of knowledge and practices among Italian pediatricians

**DOI:** 10.1186/s13052-024-01724-8

**Published:** 2024-08-19

**Authors:** Danilo Buonsenso, Manuela Ceccarelli, Bettina Camara, Donatella Angelone, Valentina Burzio, Simona La Placa, Piero Valentini, on behalf of GLNBM-SIP* and GLMM-SIMM**

**Affiliations:** 1 Department of Woman and Child Health and Public Health, Fondazione Policlinico Universitario A. Gemelli IRCCS, Largo Gemelli 89, Rome, 00168 Italy; 2 Gruppo di Studio del Gruppo di Lavoro Nazionale per il Bambino Migrante - Società Italiana di Pediatria, Rome, Italy; 3 Department of Medicine and Surgery, Unit of Infectious Diseases, University of Enna, Enna, 94100 Italy; 4 Medicine and Surgery, Università Cattolica del Sacro Cuore, Roma, Italia; 5 Department of Emergency and General Pediatrics, Pediatric Emergency Unit, Bambino Gesù Childrens Hospital, IRCCS, Rome, Italy; 6 Azienda Ospedaliera Universitario, SC di Pediatria, Ospedale Maggiore della Carità di Novara, Novara, Italia; 7 Azienda Sanitaria Provinciale di Trapani, UOC di Neonatologia e TIN, Trapani, Italia

**Keywords:** Age assessment, Migrant children, Wrist ultrasound, Multidisciplinary

## Abstract

**Background:**

Increases in migration patterns in the recent years have led to a continuously growing number of unaccompanied foreign minors (UFMs) entering Italy. As part of processing and integration, age assessment is performed by pediatricians upon request of regulatory bodies. Updated guidelines for age estimation procedures were published in 2020 in order to prioritize the well-being of the minors and the accuracy of the assessment. Nonetheless, literature suggests that the recently established multidisciplinary approach has not yet been widely adopted by physicians.

**Methods:**

A cross-sectional exploratory survey was distributed to pediatricians in Italy in order to gauge their range of experience with UFMs and age assessment protocols.

**Results:**

In total 344 pediatricians participated in the survey, originating from varied regions in Italy. Out of pediatricians who reported conducting age assessment procedures (38.9%), only a small fraction (14.2%) confirmed being knowledgeable about the methodology. Instead, a significant portion (28.8% and 56.4%) either had partial awareness or lacked knowledge of these procedures. These responses significantly differed when comparing hospital and outpatient pediatricians or according to their geographical area of work (*p* <0.05).

**Conclusion:**

Survey responses suggest that a gap in awareness and experience regarding a multidisciplinary approach to age estimations still exists, likely in part due to a lack of resources, especially at the regional level. In the future, efforts towards the education of professionals and mobilization of resources for investment in the field will be crucial for the improvement of work with UFMs and other migrant populations.

**Supplementary Information:**

The online version contains supplementary material available at 10.1186/s13052-024-01724-8.

## Introduction

The number of migrant and refugee children arriving in Italy has seen a rapid increase since 2020, a large proportion of those children being unaccompanied or separated from their parental figures [1]. In the first half of 2023, more than 6.000 minors arrived in Italy unaccompanied, an increase of more than double compared to the previous year [2]. A considerable percentage of arriving individuals lack identifying documentation, which hinders access to support services and protections for incoming migrants (https://www.bbc.com/news/world-europe-62283202) [3]. It becomes imperative that identity is readily established to its full extent, particularly in the case of children. Undocumented migrant children have decreased rates of enrolment in schools [4], social programs [5], and more limited access to medical care such as vaccinations and mental health services [6, 7].

Age assessment is essential in the process of identity determination so children may benefit from access to dedicated programs and care [8]. The incorrect assessment of age for incoming migrants can lead children to being placed in unsafe environments, such as adult accommodations and detention, and keeps them from accessing the social programs and protections dedicated to their specific age groups [9]. Current protocols recommended by the European Asylum Support Office (EASO) state that under regulations that involve taking into consideration the child’s best interest, age assessment should be conducted only if deemed appropriate [10]. Age determination has large margins for error and relevant variables can be misleading, especially if the child has undergone puberty [11]. A margin of up to 5 years can be reasonably expected [12] due to factors like ethnicity, nutrition, and history of disease [13].

According to the EASO, a non-medical approach should be prioritized, which encompasses interviews and the assessment of available evidence, granting the individual the benefit of the doubt [14]. Should medical methods be utilized, radiation free approaches should be applied prior to X-Ray-based testing [14]. Medically-conducted age assessment tests rely on factors such as valuation of sexual maturity (i.e. Tanner Staging [15]), height, weight, skin rating, radiological imaging to assess fusing of carpal bones, development of molars, clavicle fusion, as well as imaging of knees and hands [16].

Age determination is still, for the most part, performed according to regional practices and is not regulated by an international system [17]. X-Ray imaging has been widely used as the primary medical approach, with most countries in the European Union relying on carpal bone fusion analysis for age estimations according to the Greulich and Pyle reference or the Tanner-Whitehouse method [18, 19]. Reliance on this technique alone is, however, subject to significant margins of error, and inexact probability distributions [19]. Previous studies have indicated that a large proportion of Italian pediatricians are not applying a multifactorial approach to age determination [20]. Therefore, the aim of this study is to assess the general understanding of age assessment practices in the Italian pediatrician community, and determine the awareness of diverse testing methods, and to map their previous experiences in the field.

## Methodology

A cross-sectional exploratory survey was conducted with the objective of assessing the knowledge basis of pediatricians across various Italian regions regarding work with unaccompanied foreign minors (UFMs) and standard age-estimation procedures. The question form (see Appendix 1) was developed according to the investigation objectives, and was maintained within the scope of an exploratory survey. The survey was prepared by pediatricians expert in the care of Migrant Children that are active members of the Italian Working Group for the Migrant Child (Gruppo di Lavoro Nazionale per il Bambino Migrante-GLNBM) of the Italian Society of Pediatrics (Società Italiana di Pediatria -SIP). Ten members of the GLNBM initially met virtually to discuss the topic and organize a first draft of questions. Then, the initial draft was uploaded on a shared online platform and three rounds of revisions and comments were done, leading to the final version of the survey. The survey was eventually uploaded on a Google form and a survey link was generated. The first question also included a consent to participate and share the released information, as well as the use of the information for the writing of a scientific report.

Included participants were actively-practicing pediatric physicians in Italy with an ongoing active registration within the Italian Society of Pediatrics (- SIP). The society has about 10,000 subscribers including both family (outpatient) and hospital/academic pediatricians.

Pediatric physicians, both regional and associated with hospitals, received the survey via the web-link. The study was conducted from February 9th, 2024, to October 9th, 2024. Participants were contacted by e-mail directly from the Italian Society of Pediatrics administrative team that emailed all the active subscribers twice, three weeks apart. A total of 12 questions were included in the form, as well as 5 questions for demographic determination (Appendix 1). The original survey was conducted in Italian, and the English translation is provided in Appendix 2. Participants were presented with 3 or more answer choices to every question, as well as “N/A” as an option not to answer. Options included specifics regarding age assessment practice and experience working with under-age unaccompanied migrants, and multiple answers were allowed on selected questions. Analysis was conducted by excluding missing or “N/A” responses, and all data were evaluated as percentages (%). Total number of answers are indicated for every question, and *p*-values were calculated for significance analysis. A *p-*value of less than 0.05 was deemed statistically significant. Results are displayed on Tables 1 and 2.

**Table 1:** Inferential analysis by work setting

		**Hospital Pediatrician**	**Regional Pediatrician**	***p***-value
**Sex (*n* = 344)**	*Female*	135 (39.2)	109 (31.7)	0.281
	*Male*	52 (15.1)	46 (13.4)	
	*I would rather not reply*	0 (0.0)	2 (0.6)	
**Age (*n* = 344)**	*25–30*	25 (7.3)	0 (0.0)	** < 0.001**
	*31–40*	52 (15.1)	19 (5.5)	
	*41–50*	26 (7.6)	33 (9.6)	
	*51–65*	59 (17.2)	52 (15.1)	
	> *65*	25 (7.3)	53 (15.4)	
**Area (*n* = 343)**	*North*	99 (28.9)	91 (26.5)	0.297
	*Center*	38 (11.1)	36 (10.5)	
	*South*	49 (14.3)	30 (8.7)	
**Question n. 1 (*n* = 341)**	*Never*	42 (12.3)	97 (28.4)	** < 0.001**
	*Sometimes*	118 (34.6)	45 (13.2)	
	*Often*	27 (7.9)	12 (3.5)	
**Question n. 2 (*n* = 342)**	*No*	97 (28.4)	97 (28.4)	0.157
	*Yes*	28 (8.2)	21 (6.1)	
	*Some*	61 (17.8)	38 (11.1)	
**Question n. 3 (*n* = 343)**	*No*	85 (24.8)	117 (34.1)	** < 0.001**
	*Yes*	97 (28.3)	37 (10.8)	
	*I do not remember*	5 (1.5)	2 (0.6)	
Question n. 4* (*n* = 142)	*Radiologic tests*	85 (59.9)	32 (22.5)	0.640
	*Clinical examination*	64 (45.1)	20 (14.1)	
	*Pediatric auxologic evaluation*	55 (38.7)	22 (15.5)	
	*Psychological evaluation*	11 (7.7)	6 (4.2)	
	*Neuropsychiatric evaluation*	7 (4.9)	5 (3.5)	
**Question n. 5 (*n* = 135)**	*No*	40 (29.6)	10 (7.4)	**0.006**
	*Yes*	53 (39.2)	18 (13.3)	
	*I do not remember*	5 (3.7)	9 (6.7)	
**Question n. 6 (*n* = 264)**	*Police headquarters*	37 (14.0)	14 (5.3)	0.827
	*Public prosecutor office at juvenile court*	19 (7.2)	6 (2.3)	
	*Reception center*	11 (4.2)	4 (1.5)	
	*Prefecture*	2 (0.8)	2 (0.8)	
	*Other*	10 (3.8)	6 (2.3)	
	*I do not remember*	16 (6.1)	5 (1.9)	
**Question n. 7 (*n* = 135)**	*It was the only one I could carry out*	12 (8.9)	5 (3.7)	0.295
	*It was the only one I knew*	16 (11.8)	8 (5.9)	
	*It was the best one I could choose*	52 (38.5)	23 (17.0)	
	*It was the one I was requested to carry out*	17 (12.6)	2 (1.5)	
**Question n. 8 (*n* = 343)**	*No, there is not*	9 (2.6)	1 (0.3)	0.072
	*I do not know of it*	145 (42.3)	127 (37.0)	
	*Yes, I do know of it*	33 (9.6)	28 (8.1)	
**Question n. 9 (*n* = 116)**	*No*	46 (39.6)	34 (29.3)	> 0.999
	*Yes*	21 (18.1)	15 (12.9)	
Question n. 10* (*n* = 75)	*Sequential multidisciplinary check-ups*	25 (33.3)	14 (18.7)	0.985
	*Sequential radiological check-ups*	5 (6.7)	4 (5.3)	
	*I do not know*	6 (8.0)	2 (2.7)	
	*I do not remember*	4 (5.3)	4 (5.3)	
	*None in the list*	4 (5.3)	3 (4.0)	
	*Left wrist and hand XR*	3 (4.0)	3 (4.0)	
	*Pediatric auxologic evaluation*	2 (2.7)	1 (1.3)	
	*Endocrinologic evaluation*	1 (1.3)	1 (1.3)	
	*Clinical evaluation*	1 (1.3)	0 (0.0)	
	*Medical and legal advice*	1 (1.3)	0 (0.0)	
Question n. 11* (n = 90)	*Pediatrician/Auxologist/ Endocrinologist*	52 (57.8)	24 (26.7)	0.858
	*Social worker*	35 (38.9)	16 (17.8)	
	*Psychologist/Child neuropsychiatrist*	49 (54.4)	22 (24.4)	
	*Radiologist*	24 (2.7)	10 (11.1)	
	*Other*	11 (12.2)	5 (5.6)	
	*I do not remember*	7 (7.8)	7 (7.8)	
	*None in the list*	4 (4.4)	3 (3.3)	
**Question n. 12 (*n* = 143)**	*No*	4 (2.8)	2 (1.4)	**0.048**
	*Yes*	34 (23.8)	14 (9.8)	
	*I do not know*	44 (30.8)	45 (31.5)	

Comparisons of responses according to hospital and regional pediatricans

**Table 2:** Inferential analysis by geographic area

		**North**	**Center**	**South**	***p***-value
**Sex (*n* = 343)**	*Female*	140 (40.8)	52 (15.2)	51 (14.9)	0.533
	*Male*	49 (14.3)	22 (6.4)	27 (7.9)	
	*I would rather not reply*	1 (0.3)	0 (0.0)	1 (0.3)	
**Age (*n* = 343)**	*25–30*	15 (4.4)	4 (1.2)	6 (1.7)	0.237
	*31–40*	38 (11.1)	20 (5.8)	13 (3.8)	
	*41–50*	40 (11.7)	10 (2.9)	9 (2.6)	
	*51–65*	54 (15.7)	22 (6.4)	34 (9.9)	
	> *65*	43 (12.5)	18 (5.2)	17 (5.0)	
**Question n. 1 (*n* = 340)**	*Never*	82 (24.1)	25 (7.4)	32 (9.4)	0.803
	*Sometimes*	86 (25.3)	38 (11.2)	38 (11.2)	
	*Often*	21 (6.2)	9 (2.6)	9 (2.6)	
**Question n. 2 (*n* = 342)**	*No*	102 (29.9)	37 (10.9)	55 (16.1)	**0.022**
	*Yes*	26 (7.6)	10 (2.9)	13 (3.8)	
	*Some*	61 (17.9)	26 (7.6)	11 (3.2)	
**Question n. 3 (*n* = 343)**	*No*	116 (33.9)	42 (2.3)	44 (12.9)	0.588
	*Yes*	71 (20.8)	30 (8.8)	32 (9.4)	
	*I do not remember*	2 (0.6)	2 (0.6)	3 (0.9)	
Question n. 4* (*n* = 141)	*Radiologic tests*	71 (50.3)	22 (15.6)	24 (17.0)	**0.047**
	*Clinical examination*	45 (31.9)	18 (12.8)	20 (14.2)	
	*Pediatric auxologic evaluation*	34 (24.1)	17 (12.1)	26 (18.4)	
	*Psychologic and/or neuropsychiatric evaluation*	8 (5.7)	8 (5.7)	12 (8.5)	
**Question n. 5 (*n* = 134)**	*No*	31 (23.1)	14 (10.4)	5 (3.7)	**0.033**
	*Yes*	33 (24.6)	12 (9.0)	25 (18.7)	
	*I do not remember*	7 (5.2)	3 (2.2)	4 (3.0)	
**Question n. 6 (*n* = 131)**	*Police headquarters*	30 (22.9)	11 (8.4)	9 (6.9)	0.383
	*Public prosecutor office at juvenile court*	11 (8.4)	3 (2.3)	11 (8.4)	
	*Reception center*	9 (6.9)	2 (1.5)	4 (3.1)	
	*Prefecture*	1 (0.8)	2 (1.5)	1 (0.8)	
	*Other*	10 (7.6)	4 (3.1)	2 (1.5)	
	*I do not remember*	11 (8.4)	5 (3.8)	5 (3.8)	
**Question n. 7 (*n* = 135)**	*It was the only one I could carry out*	11 (8.2)	3 (2.2)	3 (2.2)	0.231
	*It was the only one I knew*	14 (10.4)	5 (3.7)	5 (3.7)	
	*It was the best one I could choose*	33 (24.6)	18 (13.4)	23 (17.2)	
	*It was the one I was requested to carry out*	14 (10.4)	4 (3.0)	1 (0.7)	
**Question n. 8 (*n* = 342)**	*No, there is not*	7 (2.0)	1 (0.3)	2 (0.6)	0.347
	*I do not know of it*	151 (44.2)	63 (18.4)	58 (17.0)	
	*Yes, I do know of it*	31 (9.1)	10 (2.9)	19 (5.6)	
**Question n. 9 (*n* = 115)**	*No*	44 (38.3)	19 (16.5)	17 (14.8)	0.314
	*Yes*	17 (14.8)	6 (5.2)	12 (10.4)	
Question n. 10*(*n* = 74)	*Sequential or isolated multidisciplinary check-ups*	23 (31.1)	8 (10.8)	14 (18.9)	0.933
	*Sequential or isolated radiological check-ups*	9 (12.2)	2 (2.7)	4 (5.4)	
	*I do not know/remember*	6 (8.1)	4 (5.4)	6 (8.1)	
	*None in the list*	4 (5.4)	1 (1.3)	2 (2.7)	
Question n. 11* (*n* = 89)	*Pediatrician/Child auxologist/Child endocrinologist*	40 (44.9)	17 (19.1)	20 (22.5)	0.595
	*Social worker*	25 (28.1)	8 (9.0)	17 (19.1)	
	*Psychologist/Child neuropsychiatrist*	35 (39.3)	12 (13.5)	26 (29.2)	
	*Radiologist*	21 (23.6)	7 (7.9)	5 (5.6)	
	*Other*	6 (6.7)	4 (4.5)	5 (5.6)	
	*I do not remember*	5 (5.6)	4 (4.5)	5 (5.6)	
	*None in the list*	5 (5.6)	0 (0.0)	2 (2.2)	
**Question n. 12 (*n* = 144)**	*No*	0 (0.0)	3 (2.1)	3 (2.1)	0.142
	*Yes*	25 (17.6)	9 (6.3)	13 (9.2)	
	*I do not know*	46 (32.4)	22 (15.5)	21 (14.8)	

Comparisons of responses according to geographical areas

## Results

### Demographics

A total of 344 physicians provided responses to the survey, 54.4% practicing in a hospital, while 45.6% were regional pediatricians (Appendix 3). The majority of participants were female (70.9%), the remainder being male (20.5%) or unidentified (0.6%), which was not significantly different in terms of work setting (*p-*value 0.281) or geographic area (*p-*value 0.533). The largest proportion of physicians fell within the 52–65 age range (32.3%), followed by those older than 65 (22.7%). Participants younger than 30 made up 7.3% of the study group, 20.6% aged 31–40 and 17.2% from 41 to 50. There was a statistically significant difference in age category percentage between the participants group associated with hospital settings or regional work (*p-*value < 0.001), in that most of those in the 31–40 range work in hospitals, and most of those over 65 were regional physicians (Table 1). Nonetheless, age groups did not vary significantly based on geography. Just over half of the answers came from physicians located in the north of Italy (55.2%), followed by the South and Islands (23%), then the central regions (21.5%). Specifics regarding location of the study population are displayed in Appendix 3.

### Study group experience with unaccompanied foreign minors

Across the surveyed group, 47.4% claimed to have had at least some experience with unaccompanied foreign minors (UFMs), and 11.3% worked with UFMs often. The rest of the participants (40.4%) had never experienced work with UFMs. Hospital pediatricians had significantly higher experience than regional pediatricians (*p-*value < 0.001), but no notable difference was noted based on geographic areas (*p-*value 0.803). When asked if they had previously performed age assessment procedures, 56.6% answered no, and 38.9% selected yes. Similarly, differences in response between pediatricians working regionally and those associated with hospitals was statistically significant (*p-*value < 0.001), but not between those located in the south, north and central Italy (*p-*value 0.588).

### Knowledge of age assessment protocols

Regarding age assessment procedures, 56.4% had no knowledge of age assessment procedures, 28.8% declaring to have only “some”, and 14.2% affirmed their knowledge on the matter. Hospital and regional pediatricians had insignificant differences in response (*p-*value 0.157), but participants practicing in the northern part of Italy selected “some” and “no” knowledge significantly more than those of other areas (*p-*value 0.022). Specific evaluation protocols included left wrist and hand X-rays (31.1%), clinical examination (24.4%), pediatric auxologic evaluation (22.4%), psychological evaluation (4.9%), neuropsychiatric evaluation (3.5%), orthopantomography (0.9%), and collarbone computerized tomography (0.3%). Professional setting did not seem to have an effect on response, yet percentage analysis based on geography indicated that a significant majority of radiologic tests were performed by northern region physicians (*p-*value 0.047). Cultural mediators were only present 20.6% of the time, 4.1% of physicians not recalling whether one was present during procedures. Hospital pediatricians answered both negatively and positively more than regional pediatricians (*p-*value 0.006), the same pattern seen for those in northern areas (*p-*value 0.033). Age verification was performed at the request of the police 14.8% of the time, followed by juvenile court (7.3%) then reception centers (4.4%), with no statistically significant difference based on setting (*p-*value 0.827) and geography (*p-*value 0.383).

Following past age assessments, 21.8% of the study physicians rated their estimations as “the best [they] could make”, 7% claiming that estimations practices were “the only one [they] knew”; 5.5% performed the assessment “[they] were requested to carry out” and 4.9% reported age assessment methods as “the only one [they] could carry out”. Study population distributions as seen in Tables 1 and 2 did not show significant variation in terms of assessment rating. Regional availability of UFM age verification services seemed to be largely unknown by most participants (79.1%), which was consistent across analytical demographics (*p-*values 0.295 and 0.231). Physicians aware of local services reported that most of the assessments involved sequential multifactorial check-ups (11.3%), which was also consistent across analytical demographics (*p-*values 0.985 and 0.933). Professionals involved in assessments were, according to survey responses, largely pediatricians or child auxologists (18.3%), social workers (14.5%), psychologists (11%), radiologists (9.6%) and child neuropsychiatrists (9.3%). No difference in response was noted in terms of work setting (*p-*value 0.858) or geography (*p-*value 0.595). Finally, cultural mediators were available during procedures according to 14% of responses, although 25.9% of participants claimed not to know this information. Pediatricians in hospital settings had significantly higher positive responses regarding the presence of cultural mediators when compared to regional ones (*p-*value 0.048), but geographic groups showed no notable differences (*p-*value 0.142).

## Discussion

In this study, we found that hospital physicians had significantly more experience working with patients classified as unaccompanied foreign minors, when compared to pediatricians working under regional systems. This is to be expected, considering that immigrants to Italy are more likely to end up in the main urban areas, and hospitals tend to be associated with regulatory government bodies rather than local pediatric clinics (https://migrant-integration.ec.europa.eu/library-document/presence-migrants-metropolitan-cities_en). On a similar note, pediatricians in hospital settings had performed more age assessment procedures than their colleagues working in regional clinics. Notably, however, despite 38.9% of pediatricians claiming to have performed age assessment procedures in the past, only 14.2% were affirmative about their knowledge of age assessment methodology. Rather, 28.8% and 56.4% of answers regarding the awareness of procedures corresponded to “some” or “[none]”, respectively. The indication that, despite a considerable portion of participants having experience in age assessments, only a small fraction seems to be confident about their expertise on the matter, highlights a gap in the knowledge of standardized practices and protocols. Education regarding estimation of a patient’s age varies highly amongst medical specializations and expertise; pediatricians may be more aware of physical development milestones, while radiologists are versed in the identification of radio-imaging reference points. It is this variation however, that can lead to different choices of methodology for physicians assigned with estimating the age of an undocumented foreign minor. It is not unexpected, then, that wrist and hand x-rays were the methods most commonly used in estimations, followed by clinical and auxology evaluations.

Literature has suggested that the use of a multifactorial approach to age assessment is more accurate than the analysis of single sites, in both male and female subjects [21]. A combination of magnetic resonance imaging (MRI) analysis of clavicles, wrists and molars, as well as sexual and anthropometric maturation data has been shown to have decreased absolute error margins compared to single-site approaches, correctly classifying male and female individuals as minors in 90% and 91% of cases, respectively [21]. Italian legislation passed in 2017 introduced changes to age assessment procedures with the objective of protecting unaccompanied minors; it states that assessment may be done only if reasonable doubts exist regarding the individual’s alleged age [22]. More recently, in 2020, the “Multidisciplinary protocol for determining the age of unaccompanied foreign minors” was published by the Italian National Health System (INHS), indicating that multiple interdisciplinary professionals should be involved in the process of age estimation, using a multi-step approach [22–24].

Despite the established INHS guidelines for a multidisciplinary approach to age determination, the results of this survey seem to confirm the notion that pediatricians are largely unaware of these guidelines. The gap in information seems to range from methodological approaches, to knowledge of local assessment services, as well as the presence of necessary professionals such as cultural mediators, especially in the regional contexts. It is imperative to highlight however, that in most settings, underfunded and under-equipped facilities account for the lack of proper protocol. This emphasizes not only the need for educational strategies, but also a push for regional funding and development. Northern regions, in particular, seemed to rely more heavily on radiology for age assessments, which could partially result from facilitated access to equipment [25], but could also lead to a reliance on those methods. A preference for X-ray and radiology-based methods can arise from the seemingly objective nature of developmental markers, which has been argued to be vastly unreliable [26]. A lack of local resources may also discourage the involvement of social workers and cultural mediators, which have been recommended by the INHS. Southern regions of Italy have especially been affected by a lack of monetary incentives, straining the system through which age assessment procedures would take place [27, 28].

The reliance on X-ray and radiology methods for age assessment has been shown to have significant consequences that can be highly impactful to the safety and social development of the unaccompanied foreign minors. Variation in bone development occurs even amongst individuals of the same age, and can be caused by genetic factors, nutrition, and history of disease [29]. The specific bones that are targeted in age estimation tests—such as the wrist and hands—are also not always reliable representations of physical development depending on the physician’s level of experience on the matter [30]. Imaging also fails to consider social history, health background, personal maturity levels, and the magnitude of factors that may sway final estimations of a minor’s age. The psychological impact of invasive procedures in newly-arrived, vulnerable minors should also not be discounted. The wrongful detention of self-proclaimed minors migrating unaccompanied as a result of improper age determination procedures has been documented in the past [31], demonstrating the significant consequences of failures to carry-out appropriate conduct and engage the necessary resources.

A survey of Local Health Authorities (ASL) in Italy was conducted in 2022 by the INMP (Istituto Nazionale per la promozione della salute delle popolazioni Migranti ed il contrasto delle malattie della Povertà) to evaluate the implementation of the 2020 guidelines regarding age assessment published by the INHS [20]. The results showed that the multidisciplinary approach was still not widely implemented at a local level, with particular emphasis on the lack of professional teams ready to fulfill the varying roles as part of the physical, psychological and social aspects of age estimations. It was also noted that a lack of concrete communication channels and practices between authorities involved in the process—such as the courts, health authorities and law enforcement—was likely a contributing factor in the delay of protocol reform. As suggested by the INHS [22], age determination procedures should involve a multi-step approach involving the expertise of professionals across disciplines. It becomes imperative then, that improvements to age assessment practices in Italy involve the participation of multiple governing bodies and institutions. The promotion of adequate training, mobilization of resources, and the incentivisation of research in development should therefore be a part of future efforts in the field.

The main limitation of this study is intrinsic in its design, being a survey. Also, a relatively small number of Italian pediatricians provided their responses, although there was good national coverage from Northern to Southern areas. Strength of this study is the national coverage, the inclusion of pediatricians working both in Hospital and outpatient settings, and the first detailed investigation of current practices and knowledge on the topic.

## Conclusion

Ultimately, as emphasized in the 2020 INHS guidelines, the priority should remain in the best interest of the unaccompanied minors, considering the significant impact age assessments would have on arriving migrants. The practice of a “benefit of the doubt” approach and the avoidance of unnecessary procedures are to be placed at the forefront of decision making regarding young foreigners, as well as the acknowledgement and understanding that no single method is able to precisely determine the age of an individual. It should be taken into consideration as well, that current set-back are, in large part, caused by lack of funding and resource acquisition for systems and professionals involved in the process of migrant reception and integration. Future endeavors should consider the investment of resources towards the development of less intrusive methods for age estimations, and research into the improvement of the efficiency and accuracy of current ones.

### Supplementary Information

Supplementary Material 1

Supplementary Material 2

## Data Availability

Available upon request to the corresponding author.
