# Supplementary material for: Age assessment of unaccompanied foreign minors: an analyses of knowledge and practices among Italian pediatricians
Source: Ital J Pediatr. 2024 Aug 19;50:151. doi: 10.1186/s13052-024-01724-8 (PMC11331759; doi:10.1186/s13052-024-01724-8)
Supplement: Supplementary file 2 — Supplementary Material 2 [file 13052_2024_1724_MOESM2_ESM.docx]

**SUPPLEMENTARY MATERIAL**

**Appendix 1**

**Original Survey:**

Indagine sulle metodiche di valutazione dell’età di presunti minorenni senza documentazione.

Gentile Collega,

Chiediamo la Sua cortese partecipazione a questa indagine sulle metodiche di valutazione dell’età di presunti minorenni ma senza documentazione che lo attesti, finalizzata a tracciare le pratiche attualmente in atto in Italia(anche i protocolli e linee guida)e la conoscenza che ne hanno i pediatri. L’indagine richiede non più di 1-2 minuti e ci aiuterà a mappare la situazione Italiana da cui partire per implementare future aree di intervento e formazione.

- Genere della persona che sta rispondendo:
- Uomo
- Donna
- Preferisco non rispondere
- Età
- 25-30
- 31-40
- 41-50
- 51-65
- >65
- Tipologia della sua attività lavorativa:
- Pediatria di libera scelta
- Pediatra ospedaliero
- Pediatra universitario
- Pediatria presso strutture del territorio
- Pediatria presso centri di accoglienza per migranti
- Pediatra in ambulatorio a bassa soglia di accesso per migranti
- Other:
- Regione di lavoro:
- Regione Abruzzo
- Regione Basilicata
- Regione Calabria
- Regione Campania
- Regione Emilia Romagna
- Regione Friuli Venezia Giulia
- Regione Lazio
- Regione Liguria
- Regione Lombardia
- Regione Marche
- Regione Molise
- Regione Piemonte
- Regione Puglia
- Regione Sardegna
- Regione Sicilia
- Regione Toscana
- Regione Trentino Alto Adige
- Regione Umbria
- Regione Val d'Aosta
- Regione Veneto
- Provincia autonoma di Trento
- Provincia autonoma di Bolzano

1. Ha mai avuto a che fare, in contesto lavorativo, con minori stranieri non accompagnati (MSNA)?

- Spesso
- Mai
- Qualche volta

1. È a conoscenza di protocolli e/o LG nazionali e internazionali riguardanti le procedure per l’accertamento dell’età dei minori non accompagnati?

- Si
- No
- Solo parzialmente.
- Se sì, quali? (risposta libera)

1. Le è mai capitato di essere attivamente coinvolto nella valutazione dell’età di presunti minorenni la cui data di nascita non è documentabile?

- Si
- No
- Non ricordo

1. Se ha risposto sì alla domanda precedente, quali di queste valutazioni hai eseguito/richiesto?

- Valutazione clinica o visita pediatrica generica
- Visita auxologica pediatrica
- Valutazione psicologica
- Valutazione neuropsichiatrica
- Rx del polso e mano sinistra
- RM del polso e mano sinistra
- TC del polso
- TC della clavicola
- TC di altro segmento osseo
- RM di altro segmento osseo
- Ortopantomografia
- Non ricordo
- Nessuna delle precedenti
- NON APPLICABILE (mai coinvolto)

1. Se ha risposto si alla domanda precedente, la valutazione è stata effettuata con l’ausilio di un mediatore culturale?

- Si
- No
- Non ricordo
- NON APPLICABILE (mai coinvolto)

1. Se ha risposto si alla domanda circa il suo coinvolgimento attivo in un caso, la richiesta di accertamento e determinazione dell’età del minore non accompagnato è stata inoltrata da parte della:

- Procura della Repubblica presso il Tribunale per i minori
- Questura
- Prefettura
- Centro di accoglienza
- Altro
- Non ricordo
- NON APPLICABILE (mai coinvolto)

1. Se sei stato coinvolto attivamente in un caso, Ritieni che la tua decisione sia stata:

- La migliore che potevi prendere
- L’unica che potevi attuare
- L’unica che conoscevi
- Quella che mi è stata richiesta
- NON APPLICABILE (mai coinvolto)

1. Lei è a conoscenza dell’esistenza, nella sua regione, di un centro/servizio che si occupa della valutazione multidisciplinare dell’età di presunti minorenni la cui data di nascita non è documentabile?

- Si, so che esiste
- No, non esiste
- Non ne sono a conoscenza

1. Se è a conoscenza, nella sua regione, di un centro/servizio multidisciplinare che si occupa della valutazione dell’età di presunti minorenni la cui data di nascita non è documentabile, sa quali sono le strategie di intervento attuate?

- Si
- No
- Non so
- NON APPLICABILE (non a conoscenza)

1. Se ne è a conoscenza, quali valutazioni vengono applicate?

- Approccio multidisciplinare sequenziale (colloquio sociale, valutazione NPI/psicologica, valutazione auxologica pediatrica, accertamenti strumentali radiografici)
- Accertamenti radiologici sequenziali (Rx del polso e mano sinistra, TC della clavicola, Ortopantomogarfia)
- Colloquio sociale
- Visita psicologica e/o NPI
- Visita auxologica pediatrica
- Rx del polso e mano sinistra
- TC della clavicola
- Ortopantomografia
- Consulenza medico-legale
- RM del polso
- TAC del polso
- TAC della clavicola
- TAC di altro segmento osseo
- RM di altro segmento osseo
- Valutazione clinica o visita pediatrica generica
- Valutazione psicologica
- Valutazione neuropsichiatrica
- Valutazione endocrinologica
- Non ricordo
- Nessuna delle precedenti
- Other:

1. Se ne è a conoscenza, quali figure professionali vengono coinvolte nella valutazione multidisciplinare?

- Assistente sociale
- Psicologo
- Neuropsichiatra infantile
- Pediatra
- Pediatra con competenze auxologiche
- Radiologo
- Endocrinologo
- Odontoiatra
- Medico-legale
- Non ricordo
- Nessuna delle precedenti
- Other:

1. Se lei è a conoscenza dell’esistenza, nella sua regione, di un centro/servizio che si occupa della valutazione multidisciplinare dell’età di presunti minorenni la cui data di nascita non è documentabile, nel centro c’è la possibilità di avvalersi del supporto di un mediatore culturale?

- Si
- No
- Non ne sono a conoscenza
- NON APPLICABILE

**Appendix 2**

**Survey Translation:**

Investigation into methods for assessing the age of presumed minors without documentation

Dear Colleague,

We ask for your kind participation in this investigation on the methods of assessing the age of presumed minors but without documentation attesting to it, aimed at tracing the practices currently in place in Italy (including the protocols and guidelines) and the knowledge of the pediatricians. The survey takes no more than 1-2 minutes and will help us map the Italian situation from which to start to implement future areas of intervention and training.

- Gender of the person who is responding:
- Man
- Woman
- Rather not answer
- Age
- 25-30
- 31-40
- 41-50
- 51-65
- >65
- Type of your work activity:
- Free choice pediatrics
- Hospital pediatrician
- University pediatrician
- Pediatrics at local facilities
- Pediatrics at reception centers for migrants
- Pediatrician in clinic with low access threshold for migrants
- Others:
- Working region:
- Abruzzo region
- Basilicata Region
- Calabria region
- Campania region
- Emilia Romagna region
- Friuli Venezia Giulia Region
- Lazio region
- Liguria region
- Lombardy region
- Marche region
- Molise region
- Piemonte region
- Puglia region
- Sardinia region
- Sicily region
- Tuscany region
- Trentino Alto Adige Region
- Umbria region
- Val d'Aosta region
- Veneto region
- Autonomous Province of Trento
- Autonomous Province of Bolzano

1. Have you ever had to deal with unaccompanied foreign minors (UFMs) in a work context?

- Often
- Never
- Sometimes

1. Are you aware of national and international protocols and/or guidelines regarding the procedures for ascertaining the age of unaccompanied minors?

- Yes
- No
- Only partially
- If yes, which ones? (free response)

1. Have you ever been actively involved in assessing the age of presumed minors whose date of birth cannot be documented?

- Yes
- No
- I do not remember

1. If you answered yes to the previous question, which of these assessments did you perform/request?

- Clinical evaluation or general pediatric visit
- Pediatric auxological visit
- Psychological evaluation
- Neuropsychiatric evaluation
- X-ray of the left wrist and hand
- MRI of the left wrist and hand
- CT of the wrist
- CT of the clavicle
- CT of another bone segment
- MRI of another bone segment
- Orthopantomography
- I do not remember
- None of the above
- NOT APPLICABLE (never involved)

1. If you answered yes to the previous question, was the evaluation carried out with the help of a cultural mediator?

- Yes
- No
- I do not remember
- NOT APPLICABLE (never involved)

1. If you answered yes to the question about your active involvement in a case, the request for verification and determination of the age of the unaccompanied minor was requested by:

- Public Prosecutor's Office at the Juvenile Court
- Precinct
- Prefecture
- Reception center
- Other
- I do not remember
- NOT APPLICABLE (never involved)

1. If you were actively involved in a case, you believe your decision was:

- The best you could get
- The only one you could implement
- The only one you knew
- The one that was requested of me
- NOT APPLICABLE (never involved)

1. Are you aware of the existence, in your region, of a center/service that deals with the multidisciplinary assessment of the age of presumed minors whose date of birth is not documented?

- Yes, I know it exists
- No, it doesn't exist
- I'm not aware of it

1. Are you aware, in your region, of a multidisciplinary center/service that deals with the assessment of the age of presumed minors whose date of birth cannot be documented, and do you know what intervention strategies have been implemented?

- Yes
- No
- I don't know
- NOT APPLICABLE (not aware)

1. If you are aware, what assessments are applied?

- Sequential multidisciplinary approach (social interview, NPI/psychological evaluation, pediatric audiological evaluation, instrumental radiographic assessments)
- Sequential radiological tests (X-ray of the left wrist and hand, CT of the clavicle, Orthopantomogarphy)
- Social conversation
- Psychological visit and/or NPI
- Pediatric auxological visit
- X-ray of the left wrist and hand
- CT of the clavicle
- Orthopantomography
- Medico-legal consultancy
- MRI of the wrist
- CT scan of the wrist
- CT scan of the clavicle
- CT scan of another bone segment
- MRI of another bone segment
- Clinical evaluation or general pediatric visit
- Psychological evaluation
- Neuropsychiatric evaluation
- Endocrinological evaluation
- I do not remember
- None of the above
- Others:

1. If you are aware, which professional figures are involved in the multidisciplinary evaluation?

- Social worker
- Psychologist
- Child neuropsychiatrist
- Pediatrician
- Pediatric Audiologist
- Radiologist
- Endocrinologist
- Dentist
- Coroner
- I do not remember
- None of the above
- Others:

1. If you are aware of the existence, in your region, of a center/service that deals with the multidisciplinary assessment of the age of presumed minors whose date of birth is not documented, is there the possibility of employing the support of a cultural mediator?

- Yes
- No
- I'm not aware of it
- NOT APPLICABLE

**Appendix 3**

Descriptive statistics

Sex

|  | Count | Percentage |
| --- | --- | --- |
| Female | 244 | 70.9 |
| Male | 98 | 20.5 |
| I would rather not reply | 2 | 0.6 |
| Total | 344 | 100.0 |

Age

|  | Count | Percentage |
| --- | --- | --- |
| 25-30 | 25 | 7.3 |
| 31-40 | 71 | 20.6 |
| 41-50 | 59 | 17.2 |
| 51-65 | 111 | 32.3 |
| >65 | 78 | 22.7 |
| Total | 344 | 100.0 |

Job

|  | Count | Percentage |
| --- | --- | --- |
| Hospital pediatrician | 187 | 54.4 |
| Pediatrician of territory* | 157 | 45.6 |
| Total | 344 | 100.0 |

*non sono molto convinta di questa traduzione

Region

|  | Count | Percentage |
| --- | --- | --- |
| Lombardy | 75 | 21.8 |
| Lazio | 39 | 11.3 |
| Veneto | 38 | 11.0 |
| Emilia Romagna | 37 | 10.8 |
| Campania | 24 | 7.0 |
| Toscana | 20 | 5.8 |
| Piemonte | 20 | 5.8 |
| Puglia | 19 | 5.5 |
| Sicilia | 16 | 4.7 |
| Sardinia | 11 | 3.2 |
| Liguria | 9 | 2.6 |
| Friuli Venezia Giulia | 8 | 2.3 |
| Abruzzo | 6 | 1.7 |
| Umbria | 5 | 1.5 |
| Basilicata | 5 | 1.5 |
| Marche | 4 | 1.2 |
| Calabria | 4 | 1.2 |
| Trentino Alto Adige | 3 | 0.9 |
| Unknown | 1 | 0.3 |
| Total | 344 | 100.0 |

Area

|  | Count | Percentage |
| --- | --- | --- |
| North | 190 | 55.2 |
| Center | 74 | 21.5 |
| South and Islands | 79 | 23.0 |
| N/A | 1 | 0.3 |
| Total | 344 | 100.0 |

Question n. 1 - Have you ever worked with unaccompanied foreign minors (UFM)?

|  | Count | Percentage |
| --- | --- | --- |
| Never | 139 | 40.4 |
| Sometimes | 163 | 47.4 |
| Often | 39 | 11.3 |
| N/A | 3 | 0.9 |
| Total | 344 | 100.0 |

Question n. 2 - Do you know of any procedure to verify UFMs’ age?

|  | Count | Percentage |
| --- | --- | --- |
| No | 194 | 56.4 |
| Yes | 49 | 14.2 |
| Some | 99 | 28.8 |
| N/A | 2 | 0.6 |
| Total | 344 | 100.0 |

Question n. 3 - Have you ever been involved in age verification procedures?

|  | Count | Percentage |
| --- | --- | --- |
| No | 202 | 58.7 |
| Yes | 134 | 38.9 |
| I do not remember | 7 | 2.0 |
| N/A | 1 | 0.3 |
| Total | 344 | 100.0 |

Question n. 4 - Which evaluation(s) did you perform?

|  | Count | Percentage |
| --- | --- | --- |
| Left wrist and hand XR | 107 | 31.1 |
| Clinical examination | 84 | 24.4 |
| Pediatric auxologic evaluation | 77 | 22.4 |
| Psychological evaluation | 17 | 4.9 |
| Neuropsychiatric evaluation | 12 | 3.5 |
| OPT | 9 | 0.9 |
| Collarbone CT-scan | 1 | 0.3 |
| N/A | 202 | 58.7 |
| Total | 344 | 100.0 |

Abbreviations: XR, x-ray; CT, computerized tomography; OPT, orthopantomography

Question n. 5 - Was a cultural mediator there?

|  | Count | Percentage |
| --- | --- | --- |
| No | 50 | 14.5 |
| Yes | 71 | 20.6 |
| I do not remember | 14 | 4.1 |
| N/A | 209 | 60.8 |
| Total | 344 | 100.0 |

Question n. 6 - Who did request the age verification?

|  | Count | Percentage |
| --- | --- | --- |
| Police Headquarters | 51 | 14.8 |
| Public Prosecutor Office at the Juvenile Court | 25 | 7.3 |
| I do not remember | 21 | 6.1 |
| Other | 16 | 4.7 |
| Reception center | 15 | 4.4 |
| Prefecture | 4 | 1.2 |
| N/A | 212 | 61.6 |
| Total | 344 | 100.0 |

Question n. 7 - How would you rate your decision?

|  | Count | Percentage |
| --- | --- | --- |
| It was the only one I could carry out | 17 | 4.9 |
| It was the only one I knew | 24 | 7.0 |
| It was the best one I could choose | 75 | 21.8 |
| It was the one I was requested to carry out | 19 | 5.5 |
| N/A | 209 | 60.8 |
| Total | 344 | 100.0 |

Question n. 8 - In your region, is there a service for UFMs’ age verification?

|  | Count | Percentage |
| --- | --- | --- |
| No, there is not | 10 | 2.9 |
| I do not know of it | 272 | 79.1 |
| Yes, I do know of it | 61 | 17.7 |
| N/A | 1 | 0.3 |
| Total | 344 | 100.0 |

Question n. 9 - Do you know which strategies they use?

|  | Count | Percentage |
| --- | --- | --- |
| No | 80 | 23.3 |
| Yes | 36 | 10.5 |
| N/A | 228 | 66.3 |
| Total | 344 | 100.0 |

Question n. 10 - Which evaluation(s) do they carry out?

|  | Count | Percentage |
| --- | --- | --- |
| Sequential multidisciplinary check-ups | 39 | 11.3 |
| Sequential radiological check-ups | 9 | 2.6 |
| I do not know | 8 | 2.3 |
| I do not remember | 8 | 2.3 |
| None in the list | 7 | 2.0 |
| Left wrist and hand XR | 6 | 1.7 |
| Pediatric auxologic evaluation | 3 | 0.9 |
| Endocrinologic evaluation | 2 | 0.6 |
| Clinical evaluation | 1 | 0.3 |
| Medical and legal advice | 1 | 0.3 |
| N/A | 269 | 78.2 |

Question n. 11 - Who are the professionals involved?

|  | Count | Percentage |
| --- | --- | --- |
| Pediatrician/Child auxologist | 63 | 18.3 |
| Social worker | 50 | 14.5 |
| Psychologist | 38 | 11.0 |
| Radiologist | 33 | 9.6 |
| Child neuropsychiatrist | 32 | 9.3 |
| Endocrinologist | 12 | 3.5 |
| Medical examiner | 11 | 3.2 |
| Dentist | 2 | 0.6 |
| Cultural mediator | 2 | 0.6 |
| I do not remember | 14 | 4.1 |
| None in the list | 7 | 2.0 |
| All in the list | 1 | 0.3 |
| N/A | 254 | 73.8 |

Question n. 12 - Is a cultural mediator available at the service?

|  | Count | Percentage |
| --- | --- | --- |
| No | 6 | 1.7 |
| Yes | 48 | 14.0 |
| I do not know | 89 | 25.9 |
| N/A | 201 | 58.4 |
| Total | 344 | 100.0 |
