# Supplementary material for: Age assessment of unaccompanied foreign minors: an analyses of knowledge and practices among Italian pediatricians
Source: Ital J Pediatr. 2024 Aug 19;50:151. doi: 10.1186/s13052-024-01724-8 (PMC11331759; doi:10.1186/s13052-024-01724-8)
Supplement: Supplementary file 1 — Supplementary Material 1 [file 13052_2024_1724_MOESM1_ESM.docx]

Reviewers’ Response

Dear Editor and Reviewer, thank you for your revision and support to improve our paper. Please find attached a point-by-point response to your comments.

**Reviewer reports:

Reviewer #2:**

I congratulate the researchers for their beautiful work. Suggestions for improving the article are listed below.

*Thank you very much for your support and overall appreciation of our paper.*

- The method and results sections in the abstract section do not provide sufficient preliminary information to the reader. It cannot adequately summarize the content of the study. In this respect, it is recommended that the method and results sections be rewritten in the abstract.

*Thank you very much, we corrected these sections as you suggested*

- The date range in which the study was conducted is stated in the text as "The study was conducted from February 9th, 2024, to October 9th, 2024." It is described as . Is there a historical misspelling or was the study terminated early?

*Sorry, this was a misspelling, as we are not yet in October 2024. We meant 2023*

- If there are inclusion or exclusion criteria regarding how the doctors included in the study were determined, they should be added to the article. Also, was a survey sent to all 10,000 people who are members of the association? Were 344 people who voluntarily participated and responded included in the study? Detailed information should be given in the text for this section. Were the survey forms sent with any randomization in terms of regional or study sites? In short, there is a lack of information in the article about how physicians are selected, and the methodology should be supported in this respect.

*Thank you, we agree these information are important to clarify. All pediatricians that had an ongoing registration to the SIP during the study period, without any exclusion criteria. Also, There was no randomization in terms of region or study sites*

- Is 344 physicians a sufficient number to complete the study? Has any power analysis, etc. been done in terms of numbers?

*The response rate was overall low, we agree with you and we acknowledged this in the study limitation. However, being an exploratory study and the first kind of such detailed investigation on the topic in Italy, we still find our data relevant for readers but also to the Italian Society of Pediatrics and Policymakers to implement correcting actions.*

- How did the older age of the majority of the doctors in the sample affect the course of the study, positively or negatively? To what extent do doctors have knowledge of developing technology and advanced examination procedures? These can be mentioned in the discussion section.

*This is definitely a good suggestion, thank you. We have added the following section: “Of note, a relatively higher proportion of participants were pediatricians older than 50 years of age. This age distribution of the respondents may have theoretically affected the relatively traditional approach based on wrist x-ray, which has been historically used to evaluate age. Conversely, more modern approaches requiring multidisciplinary teams have been more recently suggested by guidances and its implementation may have had a theorecial generational gap. However, also other factors may have had affected responses and knowledge of the most recent approaches for age assessment, including the subspecialty of the participating pediatricians, which was not assessed by our survey,”*

 -I think that ethics committee approval must be obtained in order to conduct the study.

*We understand your point, however the study did not include patients information but also practice performed by pediatricians, which responded in an anonymous way. As a kind of demonstration, on the very same journal we published a very similar study using the same approach, which was published without need of ethic committee approval. We have clarified this in the methods and declarations section.*

*The paper we have just published on Italian Journal of Pediatrics using the same methodology is this one:*

*Musolino AM, Tei M, De Rose C, Buonsenso D, Supino MC, Zampogna S, Staiano A, Raponi M, Amendolea A, Colacino V, Gori L, Manganaro A, Ricci R, D'Inzeo V, Grosso S, Villani A, Agostiniani R. Pediatric ultrasound practice in Italy: an exploratory survey. Ital J Pediatr. 2024 Jun 9;50(1):114. doi: 10.1186/s13052-024-01680-3. PMID: 38853266; PMCID: PMC11163714.*

Thank you very much,

Danilo Buonsenso

On behalf of coauthors
